# Validation of the Combined Biomarker for Prediction of Response to Checkpoint Inhibitor in Patients with Advanced Cancer

**DOI:** 10.3390/cancers13102316

**Published:** 2021-05-12

**Authors:** Jin-Chul Kim, You-Jeong Heo, So-Young Kang, Jeeyun Lee, Kyoung-Mee Kim

**Affiliations:** 1 Samsung Medical Center, Department of Medicine, Division of Hematology-Oncology, Sungkyunkwan University School of Medicine, Seoul 06351, Korea; jinchul.kim@inha.ac.kr (J.-C.K.); Jyunlee@skku.edu (J.L.); 2 Samsung Medical Center, The Samsung Advanced Institute for Health Sciences & Technology (SAIHST), Sungkyunkwan University School of Medicine, Seoul 06351, Korea; heo893@naver.com; 3 Samsung Medical Center, Department of Pathology and Translational Genomics, Sungkyunkwan University School of Medicine, Seoul 06351, Korea; sy500.kang@samsung.com

**Keywords:** immune checkpoint inhibitors, prediction, biomarker, cancer, PD-L1

## Abstract

**Simple Summary:**

IMAGiC model is the model consisting of four-gene and PD-L1 expression levels to predict immunotherapy response. The IMAGiC model’s predictive performance was validated in patients with several advanced tumor types in this study. The PFS and OS demonstrated significant differences between the dichotomous IMAGiC groups. IMAGiC group could be utilized as a binary biomarker for predicting response to immunotherapy regardless of TMB level or MSI status.

**Abstract:**

Although immune checkpoint inhibitors can induce durable responses in patients with multiple types of advanced cancer, only a limited number of patients have a known reliable biomarker. This study aimed to validate the IMmunotherapy Against GastrIc Cancer (IMAGiC) model, which was developed based on a previous study of four-gene and PD-L1 level, to predict immunotherapy response. We developed a clinical assay for formalin-fixed paraffin-embedded samples using quantitative real-time polymerase chain reaction to measure the expression level of the previously published four-gene set. The predictive performance was validated in a cohort of 89 patients with several advanced tumor types. The IMAGiC score was derived from tumor samples of 89 patients consisting of eight cancer types, and 73 out of 89 patients available for clinical response were analyzed with clinicopathological factors. The IMAGiC group (responder vs. non-responder) was determined with a specific value of the IMAGiC score as a cutoff, which was set by log-rank statistics for progression-free survival (PFS) divided the patients into 56 (76.7%) non-responders and 17 (23.3%) responders. Clinical responders (complete response/partial response) were higher in the IMAGiC responder group than in the non-responder group (70.6 vs. 21.4%). The median PFS of the IMAGiC responder group and non-responder was 20.8 months (95% CI 9.1-not reached) and 6.7 months (95% CI 4.9–11.1, *p* = 0.007), respectively. Among the 17 IMAGiC responders, 11 patients had tumor mutation burden-low and microsatellite-stable tumors. This study validated a predictive model based on a four-gene expression signature. Along with conventional biomarkers, our model could be useful for predicting response to immunotherapy in patients with advanced cancer.

## 1. Introduction

Immunotherapy, represented by immune checkpoint blockade, has demonstrated robust antitumor effects in treating various cancer types [1]. Since the initial approval of the cytotoxic T-lymphocyte–associated antigen 4 inhibitor ipilimumab in 2011, multiple checkpoint inhibitors have been developed and approved for multiple cancer types. Unlike conventional chemotherapeutic drugs, checkpoint inhibitors enhance the immune system to destroy cancer cells by blocking negative regulators expressed on the surface of immune or tumor cells [2]. Immunotherapy could induce a more durable response through these modes of action and had relatively fewer adverse events than conventional chemotherapy [3].

However, since the overall response rate to checkpoint blockade monotherapy was reported only in about 10–30% of patients in most types of cancer [4], substantial efforts are ongoing to define more reliable predictors of response to understand the biology of resistance to immunotherapy. Several biomarkers, such as microsatellite instability (MSI) status, programmed death-ligand 1 (PD-L1) expression, and tumor mutation burden (TMB) levels, have been extensively investigated. However, even these modalities did not fully predict the response to immunotherapy, and the proportion of patients with these markers was reported to be low in most types of tumors [5,6]. Additionally, the optimal cutoff of each modality is somewhat controversial, with various cutoff values used in several trials and studies. Therefore, there is still an unmet need to find another functional assay that could offer more transparent binary discrimination of responsiveness to immunotherapy.

One of the most recently studied biomarkers is analyzing transcriptomic features of tumors. Gene expression profiling could assess the simultaneous changes in the mRNA transcript levels of related genes. Several transcriptomic signatures have been developed to examine and predict the sensitivity or resistance to immunotherapy [7,8,9], most of which were based on inflammation or immune checkpoint pathway signature as cornerstones of each assay.

Recently, using a cohort of 21 patients with metastatic gastric cancer from the Samsung Medical Center, we developed a model named IMmunotherapy Against GastrIc Cancer (IMAGiC) score, based on the expression of a four-gene signature and PD-L1 combined positive score (CPS), that predicts response to pembrolizumab [10]. As a validation of our previous study, we analyzed the performance of the IMAGiC score by applying the model to another independent patient set of various tumor types in the current study.

## 2. Materials and Methods

### 2.1. Patients and Samples

From June 2019 to November 2020, tumor samples with clinicopathological factors were analyzed in patients who had previously received at least one immune checkpoint inhibitor at the Samsung Medical Center. Total RNAs were extracted from 10 (4-μm-thick) sections cut from each formalin-fixed paraffin-embedded tissue. From the tumor-rich areas (>20% tumor volume), RNA was isolated using the RNeasy FFPE kit (Qiagen, Hilden, Germany) according to the manufacturer’s instructions.

In our previous study, we developed the IMAGiC score model using NanoString platform (NanoString Technologies Inc., Seattle, WA, USA), which was calculated using gene expression levels of ubiquitin C-terminal hydrolase L1 (UCHL1), tyrosine kinase 2 (TYK2), protein kinase D1 (PRKD1), and armadillo repeat-containing X-Linked 1 (ARMCX1) gene and PD-L1 CPS. In this study, quantitative real-time polymerase chain reaction (qRT-PCR) was performed to validate the IMAGiC score model using cDNAs synthesized from total RNAs. PCR amplifications were performed in triplicate wells using the following conditions on a 7900 HT Sequence Detection System (Applied Biosystems, Foster City, CA, USA): 2 min at 50 °C and 10 min at 94 °C, followed by 40 two-temperature cycles of 95 °C for 15 s and 60 °C for 60 s. PD-L1 CPS was calculated by summing the number of PD-L1–stained cells (tumor cells, lymphocytes, macrophages) and dividing the result by the total number of viable tumor cells, multiplying by 100. Because we changed platform nanoString to qRT-PCR, linear regression model was reconstructed using mRNA expression levels of those four genes and the PD-L1 CPS of tissues with cancer. IMAGiC scores are obtained by multiplying the weights for each of the four gene expression levels and PD-L1 CPS from the linear regression model and adding them, with lower scores predicting higher chances of response to immunotherapy.

Medical records of patients were retrospectively gathered for age; sex; cancer type; treatment line, regimen, and number of cycles of immunotherapy; TMB; MSI status; PD-L1 CPS; expression level of each gene for calculating the IMAGiC score; and response to treatment.

### 2.2. Statistical Analysis

Statistical tests included Fisher’s exact test for two-sample tests of proportions and Wilcoxon rank-sum test for two-sample tests of continuous variables that did not follow a normal distribution. Pearson’s correlation was used to examine the association between the IMAGiC score and groups with several biomarkers for immunotherapy and calculate correlation coefficients. Log-rank statistics were used using the maxstat package for R to assess the cutoff point of the IMAGiC score for dividing patients into two categories, IMAGiC responder and non-responder. The Response Evaluation Criteria in Solid Tumors (RECIST) version 1.1 were used to assess treatment response. Progression-free survival (PFS) and overall survival (OS) were calculated from the start of treatment to the date of disease progression or death and death, respectively. The Kaplan–Meier curve method and the log-rank test by R package “survival” were used to compare PFS and OS between the IMAGiC responder and non-responder groups. Receiver operating characteristic (ROC) curve analysis was performed. The area under the curve (AUC) was calculated to evaluate the predictive performance of the IMAGiC model in checkpoint inhibitor response. Two-sided *p* values of 0.05 or lower were considered significant. R studio software (version 1.2.1335) was used for statistical analysis.

### 2.3. Validation in Another Cohort+

RNA sequencing data of patients with advanced non-small cell lung cancer treated with anti-PD-1/PD-L1 (GSE135222, *n* = 27) were selected to validate the IMAGiC score [11]. The ComBat function was used for adjusting gene expression data using the sva package because the validation data and test data had different platforms. Since there was no separate report of PD-L1 CPS in this study, we utilized the expression value of the CD274 gene, which encodes PD-L1 protein, as a substitute for PD-L1 CPS, as a measurement of PD-L1 mRNA expression using RNA sequencing is equivalent to PD-L1 expression by immunohistochemistry both analytically and clinically in predicting response to immune checkpoint inhibitor [12].

## 3. Results

### 3.1. Patient Clinicopathologic Characteristics

Tumor samples were extracted from formalin-fixed paraffin-embedded tissue from a total of 89 patients. Of these, 73 patients were available for the evaluation of response to immunotherapy. Baseline characteristics of patients with available response data according to treatment response (complete response (CR)/partial response (PR) versus stable disease/progressive disease (PD)) and all included patients are shown in Table 1. Overall, two had CR, 22 had PR, 31 had SD, and 18 had PD. The median age was 61 years, and 37 (50.7%) patients were men. Eight types of cancer were included in the analysis: cervical cancer, cholangiocarcinoma, colorectal cancer, gastric cancer, hepatocellular carcinoma, melanoma, sarcoma, and urothelial carcinoma. Among gastric cancers, there were no Epstein–Barr virus-positive tumors. Immunotherapy has been used alone or in combination with other chemotherapeutic agents. Atezolizumab, avelumab, durvalumab, nivolumab, and pembrolizumab were included, and pembrolizumab containing regimen was most frequently administered (41.1%). Among known biomarkers for checkpoint blockade treatment, MSI status and PD-L1 CPS were significantly different between clinical responders (CR/PR) and non-responders (SD/PD) groups.

### 3.2. IMAGiC Score/Group and Treatment Outcome

Initially, the optimal cutoff point of the IMAGiC score that divided patients into two groups, IMAGiC responders and non-responders, was determined by log-rank statistics.

**Figure 1:**
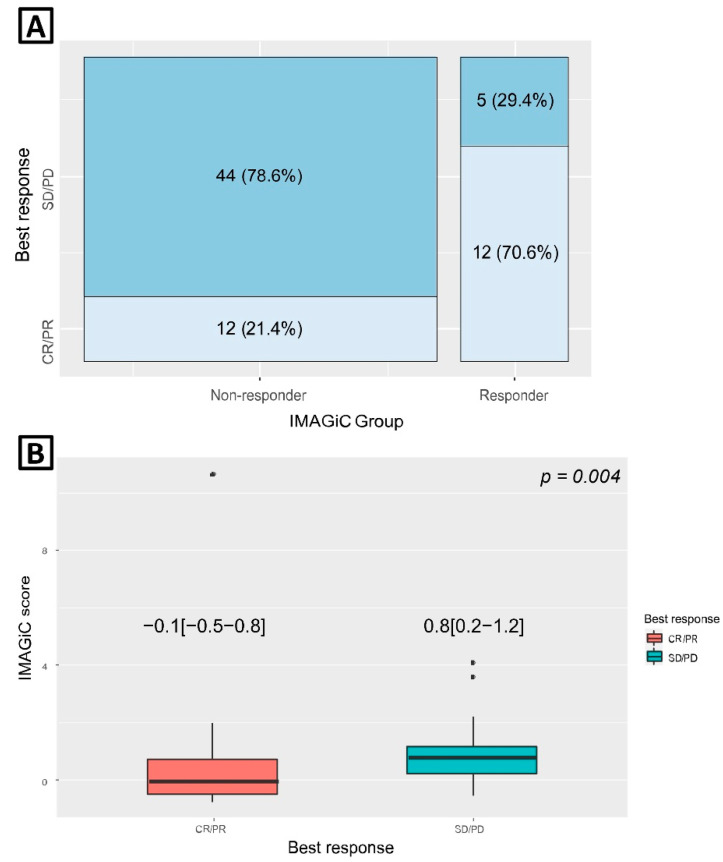
(**A**) Best overall response according to IMAGiC group. (**B**) IMAGiC score according to best overall response; expressed as median [quartile range]. CR, complete response; PR, partial response; SD, stable disease; PD, progressive disease.

**Figure 2:**
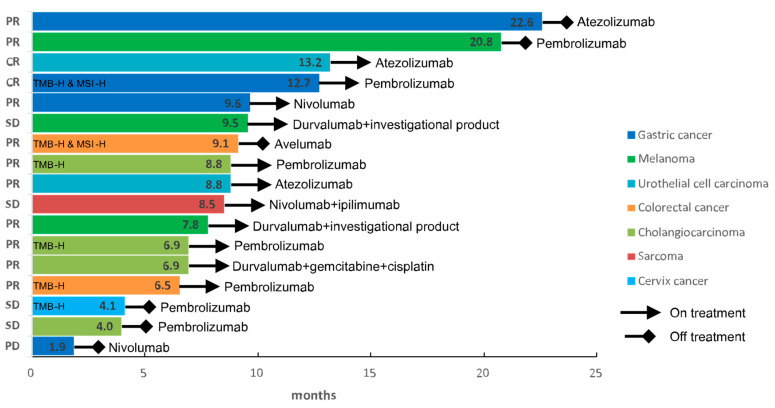
Treatment response of the patients in the IMAGiC responder group. Response indicates the best response to the immunotherapy. The number in each bar indicates progression-free survival in months. PR, partial response; CR, complete response; SD, stable disease, TMB-H, tumor mutation burden-high; MSI-H, microsatellite instability-high.

**Figure 3:**
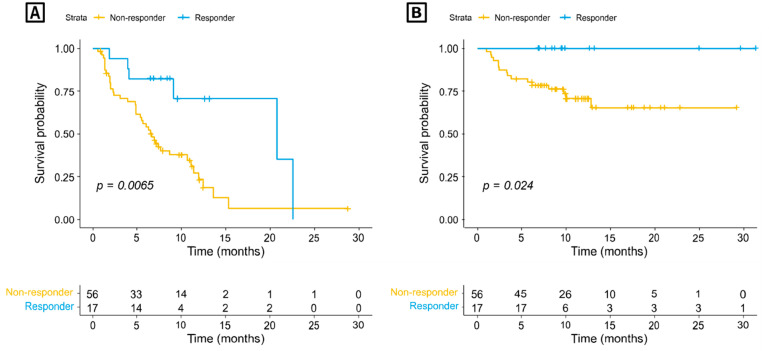
Kaplan–Meier survival curve of (**A**) progression-free survival and (**B**) overall survival according to IMAGiC non-responder/responder group.

Clinical response to immunotherapy based on the RECIST criteria was not available in 14 (20%) out of 70 IMAGiC non-responders and two (11%) out of 19 responders. The proportion of the IMAGiC responder was significantly higher in the CR/PR group than in the SD/PD group (50.0% vs. 10.2%, *p* ≤ 0.001) (Table 1). Conversely, the number of clinical responders (CR/PR) was higher in the IMAGiC responder group than in the non-responder group (70.6% versus 21.4%, Figure 1A). The IMAGiC score was also significantly different between CR/PR and SD/PD groups (Figure 1B). The treatment durations and best overall responses based on the immunotherapy regimen are shown in Figure 2. Of the 17 IMAGiC responders, two patients had MSI-high and TMB-high tumors, four others had TMB-high tumors, and the remaining 11 had TMB-low and microsatellite stable (MSS) tumors. Figure 3 demonstrates the PFS and OS data of the IMAGiC responder and non-responder groups. Kaplan–Meier survival curve analysis demonstrated that the IMAGiC responder was significantly associated with longer PFS and OS. The median PFS of responders and non-responders was 20.8 months (95% confidence interval [CI] 9.1-not reached) and 6.7 months (95% CI 4.9–11.1, *p* = 0.007), respectively. The median OS of each group was not reached but showed a clear separation between the two groups. Most events of OS analysis were censored due to a relatively short median follow-up duration of 6.9 months.

### 3.3. Association between IMAGiC Score/Group and Other Immunotherapy Biomarkers

**Figure 4:**
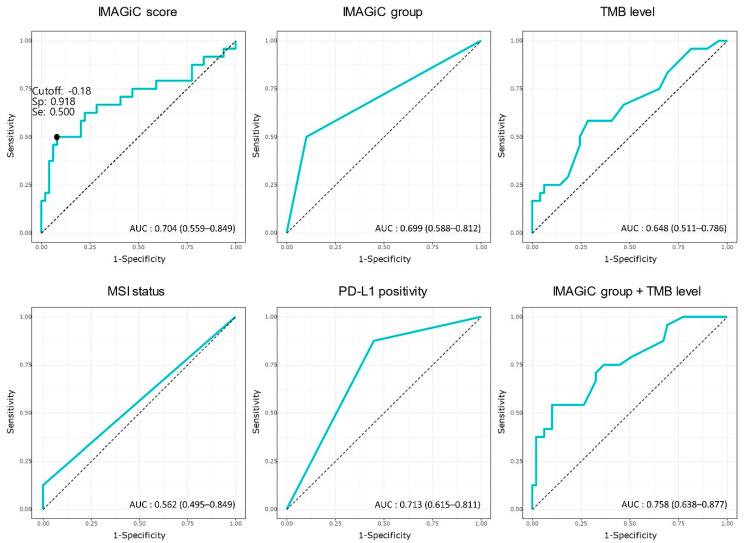
ROC curve and AUC of each biomarkers based on response to immunotherapy. TMB, tumor mutation burden; MSI, microsatellite instability; PD-L1, programmed death-ligand 1; Sp, specificity; Se, sensitivity.

**Figure 5:**
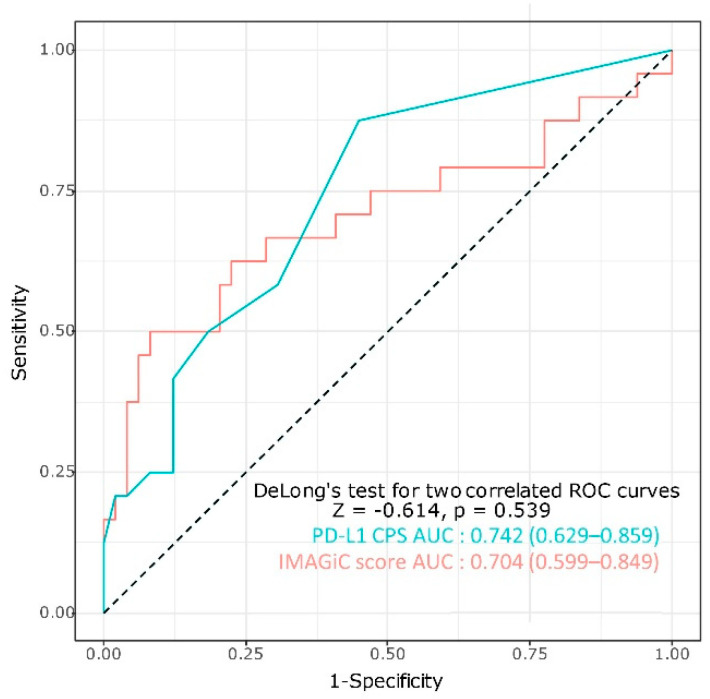
Comparison of the ROC curve of the PD-L1 CPS and the IMAGiC score based on response to immunotherapy.

The relationships between each biomarker were analyzed in a total of 89 tumor samples. In the current study, the cutoff value of the IMAGiC score was determined to be −0.18; patients with scores below this value were classified as IMAGiC responders, and those with scores above this value were classified as non-responders. IMAGiC scores were lower in TMB-high and MSI-high tumors than in TMB-low and MSS tumors (online supplemental Figure S1). However, correlation plots demonstrated a low association between the IMAGiC score and MSI status (r = 0.14) and the IMAGiC score and TMB level (r = 0.11) (online supplemental Figure S2). The PD-L1 CPS and TMB values (as a continuous variable) were significantly higher in the IMAGiC responder group than in the non-responder group. However, the TMB group (high and low, the cutoff of 10 mutations/megabase) and MSI status were not significantly different between the two groups. (online supplemental Table S1). The usefulness of the IMAGiC score and other conventional assays (TMB, MSI status, and PD-L1 CPS) as predictive biomarkers for immunotherapy was further evaluated by AUC analyses based on clinical response (CR/PR versus SD/PD) to immunotherapy (Figure 4). The AUC value of the IMAGiC score was 0.704, and the highest value was obtained for the combination of the IMAGiC score and TMB level (0.76). Additionally, the ROC curve of the PD-L1 CPS and IMAGiC score were compared using DeLong’s test for two correlated ROC curves. There was no significant difference between the two models (*p* = 0.539, Figure 5).

To validate the IMAGiC score model, we applied the IMAGiC score model for another cohort of patients with advanced non-small cell lung cancer who received immune checkpoint inhibitor treatment with published RNA sequencing data (GSE135222, *n* = 27) [11]. The AUC value for predicting clinical response to immunotherapy of the IMAGiC score, IMAGiC group, and mRNA expression of PD-L1 was 0.76, 0.69, and 0.61, respectively (online supplemental Figure S3). These results support that the IMAGiC score model might be a strong predictive biomarker to predict response for immunotherapy.

## 4. Discussion

The success of checkpoint blockade treatment in several metastatic tumors has opened a promising new opportunity for cancer therapeutics. However, only a small subset of patients respond to checkpoint inhibitors, making it crucial to identify patients who could benefit from these therapies. Current molecular testing to predict the response to immunotherapies includes panel or immunohistochemistry-based individual methods (e.g., MMR status or PD-L1 expression). However, the immune response and biology complexity renders it implausible that a single ideal biomarker could sufficiently predict immunotherapy response. In this context, we developed and evaluated the efficacy of the IMAGiC model as a predictive biomarker for advanced pan-cancer patients who received checkpoint blockade treatment. This study identified that the IMAGiC group could be utilized as a binary marker for response to immunotherapy, regardless of other conventional assays such as TMB level or MSI status.

The IMAGiC score is the value that was calculated by assigning different weights to the expression levels of each of the four genes and the PD-L1 CPS. In our study, the cutoff score, which divided patients into two categorical groups, was the point at which the separation of the survival curves for PFS in the two groups was maximized. The cutoff of the IMAGiC score was −0.18, which corresponded to the value of the highest Youden index (sensitivity plus specificity minus 1) on the ROC curve of the IMAGiC score for the prediction of clinical response (CR/PR versus SD/PD). With this cutoff value, the IMAGiC group had a predictive power with a corresponding specificity of 0.918, a sensitivity of 0.500, a positive predictive value of 0.750, and a negative predictive value of 0.790 for predicting clinical response. Therefore, it seems reasonable to set the cutoff of the IMAGiC score as −0.18 even when considering not only the PFS but also the aspect of predicting the clinical response. By employing the ROC curve, the IMAGiC score could predict the immunotherapy response (CR/PR) with an AUC of 0.704, the IMAGiC group with an AUC of 0.699, and the combination of IMAGiC group and TMB level with an AUC of 0.758, which could indicate that the conjunction of the two modalities could demonstrate better performance.

UCHL1, TYK2, PRKD1, and ARMCX1 were significantly differentially expressed between the response and the no-response group to pembrolizumab in our previous study [10]. UCHL1 was reported to promote the expression of PD-L1 through the Akt-P65 signaling pathway, and a high UCHL1 expression level inhibited T cell activity [13]. TYK2 is required for the immune response to cancer and the development of inflammation [14]. PKD1 is involved in several biological processes, including cell proliferation, migration, invasion, angiogenesis, and immune regulation [15]. The function of ARMCX1 is relatively unknown, but a recent study reported that ARMCX1 is associated with RNA modification and damage response and could be a potential prognostic marker of gastric cancer [16]. Considering each gene’s function and character, it seems valid that these genes constitute the IMAGiC score, predicting immunotherapy’s efficacy.

The results of this analysis suggest that the IMAGiC model and other modalities, including TMB level and MSI status, are moderately correlated. Although the IMAGiC score was significantly lower in the MSI-high group than in the MSS group, the number of patients with high MSI was not different between the two IMAGiC groups. Similarly, the TMB level and IMAGiC model showed a discrepant association. MSI status and TMB level, when assessed separately or in combination, could predict the clinical efficacy of immunotherapy across multiple tumor types according to previous data [17,18,19]. We found several patients whose tumors were TMB-low and MSS, which suggests that multiple approaches using several assays might be necessary for appropriate selection of patients to be treated, and our biomarker may help identify small populations predicted to show a low response rate for which immunotherapy might still be beneficial.

Recent studies have established and validated a predictive model for immunotherapy using the gene expression profiling method. Analyzing transcriptomic features of tumors could assess the simultaneous changes in the mRNA transcript levels of related genes. Several transcriptomic signatures have been developed in various tumor types to examine and predict the sensitivity or resistance to immunotherapy [7,8,9,20,21], most of which were based on inflammation or immune checkpoint pathway signature as cornerstones of each assay. In the current study, we built a model with the composition of four genes selected through the differentially expressed gene analysis and PD-L1 CPS, which were not limited to a specific pathway.

With the development of high-throughput technologies, a variety of biomarker strategies have been developed and found that multifactorial synergistic predictive markers were superior to the single marker. Comprehensive predictive biomarker models developed through integrating different types of data based on different components of tumor-host interactions is the direction of future research and will have great impacts on the field of precision immuno-oncology. Given that IMAGiC platform correlates well with high TMB in various tumor types and predicted response to checkpoint inhibitor in patients with high-MSI tumor and not responding to immunotherapy, the predictive function of the IMAGiC is thought to be at least equal or superior to PD-L1 expression. Although the superb performance of IMAGiC was not proven in the present study, possibly due to a small number of cases, it could be considered that the IMAGiC is one of the multifactorial synergistic biomarkers, and we are planning to perform a prospective clinical trial to prove this in a larger cohort.

This study has some inherent limitations. First, as we tried to construct a simplified predictive biomarker by analyzing multiple genomically heterogeneous tumors at once, it might be difficult to generalize the results due to differences in the characteristics of each cancer type. Second, the statistically determined cutoff point for the IMAGiC score in the current study would need to be validated in a generalized and larger dataset. Third, a relatively short follow-up period prevented the maturation of survival data (PFS and OS). Nevertheless, despite these drawbacks, our dichotomized IMAGiC score was identified as a robust biomarker for better predicting treatment response and improved PFS in patients treated with checkpoint blockade treatment.

## 5. Conclusions

In conclusion, our data showed the IMAGiC model’s potential as a predictive modality for immunotherapy in multiple types of advanced cancer. An appropriate cutoff for the IMAGiC responder should be determined in further large-scale studies. The application of the model to each advanced cancer type will also be further conducted.

## Figures and Tables

**Table 1:** Baseline characteristics of response evaluable patients.

Clinical Response	CR/PR	SD/PD	Total	*p*-Value
	(*n* = 24)	(*n* = 49)	(*n* = 73)	
Age (median & quartile range)	64.0 (54.0; 71.0)	59.0 (52.0; 67.0)	61.0 (52.0; 70.0)	0.466
Gender				0.457
Female	10 (41.7%)	26 (53.1%)	36 (49.3%)	
Male	14 (58.3%)	23 (46.9%)	37 (50.7%)	
Cancer type				0.107
Cervix cancer	0 (0.0%)	1 (2.0%)	1 (1.4%)	
Cholangiocarcinoma	3 (12.5%)	7 (14.3%)	10 (13.7%)	
Colorectal cancer	4 (16.7%)	0 (0.0%)	4 (5.5%)	
Gastric cancer	6 (25.0%)	13 (26.5%)	19 (26.0%)	
Hepatocellular carcinoma	0 (0.0%)	1 (2.0%)	1 (1.4%)	
Melanoma	9 (37.5%)	16 (32.7%)	25 (34.2%)	
Sarcoma	0 (0.0%)	5 (10.2%)	5 (6.8%)	
Urothelial carcinoma	2 (8.3%)	6 (12.2%)	8 (11.0%)	
Treatment line of immunotherapy				0.948
1	8 (33.3%)	15 (30.6%)	23 (31.5%)	
2	8 (33.3%)	19 (38.8%)	27 (37.0%)	
≥3	8 (33.3%)	15 (30.6%)	23 (31.5%)	
Immunotherapy regimen				0.668
Atezolizumab containing	3 (12.5%)	5 (10.3%)	8 (11.0%)	
Avelumab containing	1 (4.2%)	0 (0.0%)	1 (1.43)	
Durvalumab containing	6 (25.0%)	13 (26.4%)	19 (26.0%)	
Nivolumab containing	4 (16.7%)	11 (22.6%)	15 (20.6%)	
Pembrolizumab containing	10 (41.6%)	20 (40.7%)	30 (41.1%)	
Number of immunotherapy cycle (median & quartile range)	14.0 (11.0; 19.0)	7.0 (3.0; 9.0)	9.0 (5.0; 13.0)	<0.001
Total TMB (median & quartile range)	7.0 (4.3; 10.2)	4.7 (3.1; 7.0)	5.5 (3.1; 7.8)	0.040
TMB				0.191
High (≥10 mutations per megabase)	6 (25.0%)	6 (12.2%)	12 (16.4%)	
Low (<10 mutations per megabase)	18 (75.0%)	43 (87.8%)	61 (83.6%)	
MSI status				0.033
MSI-H	3 (12.5%)	0 (0.0%)	3 (4.1%)	
MSS	21 (87.5%)	49 (100.0%)	70 (95.9%)	
PD-L1 CPS	4.5 (1.0; 15.5)	0.0 (0.0; 3.0)	1.0 (0.0; 5.0)	0.001
IMAGiC Group				<0.001
Non-responder	12 (50.0%)	44 (89.8%)	56 (76.7%)	
Responder	12 (50.0%)	5 (10.2%)	17 (23.3%)	

CR, complete response; PR, partial response; SD, stable disease; PD, progressive disease; TMB, tumor mutation burden; MSI, microsatellite instability; MSI-H, MSI-high; MSS, microsatellite stable; PD-L1 CPS, programmed death-ligand1 combined positive score.

## Data Availability

Data are available upon reasonable request. The data that support the findings of this study are available on request from the corresponding author, K.-M.K.

## Supplementary Materials

The following are available online at https://www.mdpi.com/article/10.3390/cancers13102316/s1, Figure S1: IMAGiC score according to (A) TMB and (B) MSI status; median [quartile range]. Figure S2: Correlations between the IMAGiC model, TMB, MSI status, and PD-L1 CPS. Figure S3: ROC curve and AUC of each biomarker based on response to immunotherapy in advanced non-small cell lung cancer cohort. Table S1: Clinical characteristics and biomarker profile according to the IMAGiC responder/non-responder group.
