# Supplementary material for: Validation of the Combined Biomarker for Prediction of Response to Checkpoint Inhibitor in Patients with Advanced Cancer
Source: Cancers (Basel). 2021 May 12;13(10):2316. doi: 10.3390/cancers13102316 (PMC8151730; doi:10.3390/cancers13102316)
Supplement: Supplementary file 1 [file cancers-13-02316-s001.zip › cancers-1155891-supplementary-final -final.pdf]

Validation of the Combined Biomarker for Prediction of Response to Checkpoint Inhibitor in Patients with Advanced Cancer

Jin-Chul Kim, You-Jeong Heo, So-Young Kang, Jeeyun Lee and Kyoung-Mee Kim

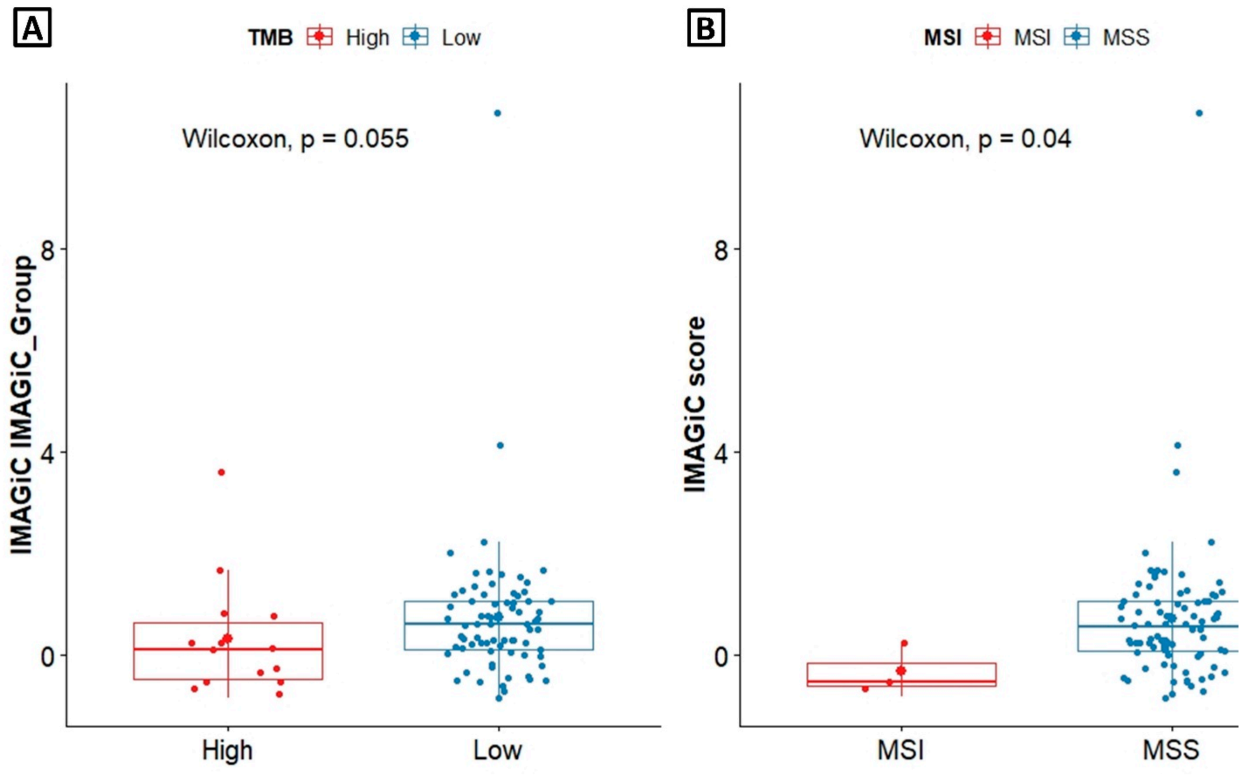

Figure S1. IMAGiC score according to (A) TMB and (B) MSI status; median [quartile range]. TMB, tumor mutation burden; MSI, microsatellite instability.

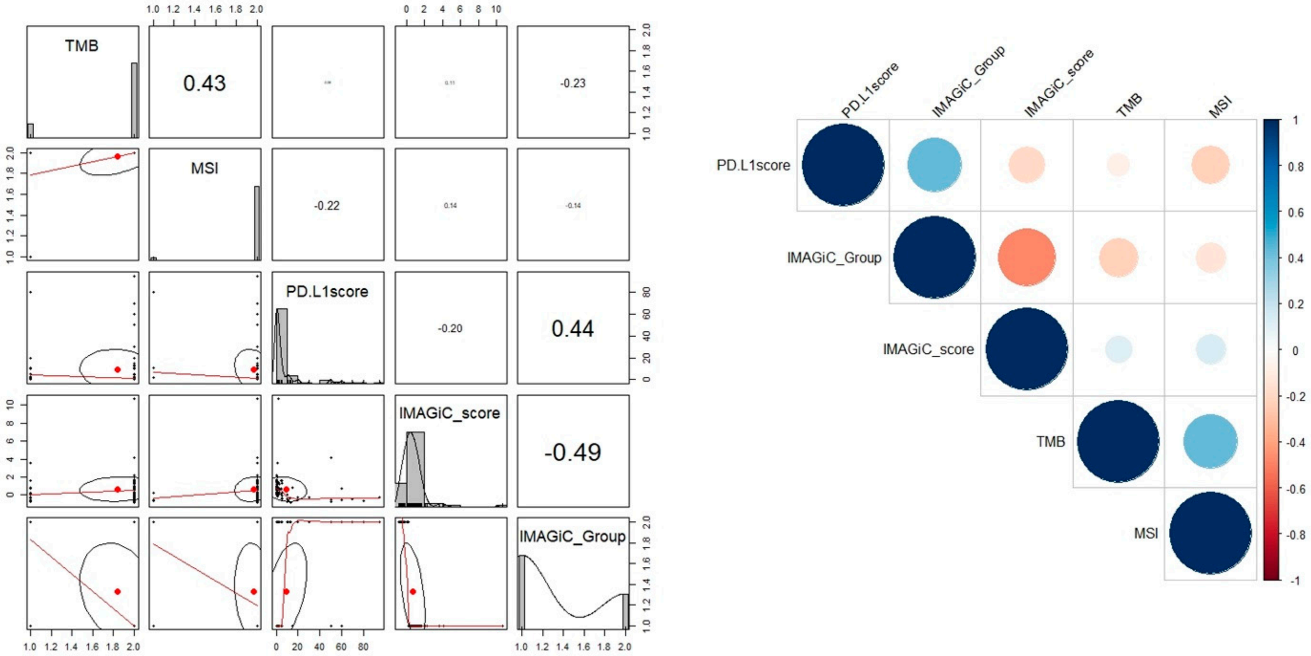

Figure S2. Correlations between the IMAGiC model, TMB, MSI status, and PD-L1 CPS. TMB, tumor mutation burden; MSI, microsatellite instability; PD-L1 CPS, programmed death-ligand 1 combined positive score.

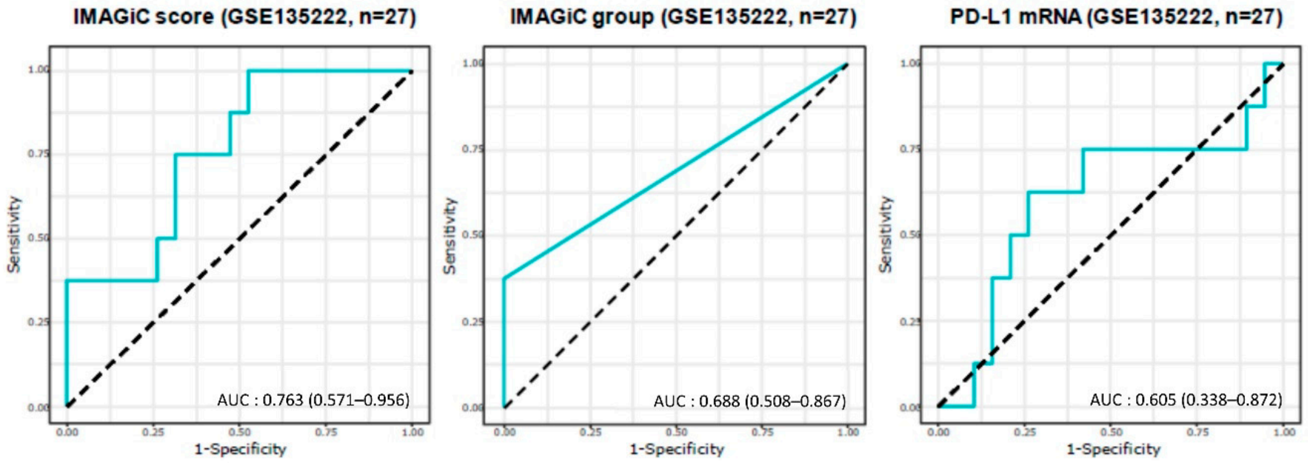

Figure S3. ROC curve and AUC of each biomarker based on response to immunotherapy in advanced non-small cell lung cancer cohort.

Table S1. Clinical characteristics and biomarker profile according to the IMAGiC responder/non-responder group.

IMAGiC Group	Non-Responder (*n* = 70)	Responder (*n* = 19)	*p*-Value
**Gender**			0.309
- Female	36 (51.4%)	10 (52.6%)	
- Male	14 (58.3%)	23 (46.9%)	
**Age** (median & quartile range)	60.5 [50.0; 69.0]	59.0 [53.5; 72.5]	0.663
**MSI status**			0.114
- MSI-H	1 (1.4%)	2 (10.5%)	
- MSS	69 (98.6%)	17 (89.5%)	
**TMB**			0.068
- High	8 (11.4%)	6 (31.6%)	
- Low	62 (88.6%)	13 (68.4%)	
**PD-L1 (CPS)** (median & quartile range)	4.5 [0.0; 2.0]	20.0 [10.5; 50.0]	<0.001
**TMB** (median & quartile range)	5.1 [3.1; 7.8]	7.0 [5.1; 12.9]	0.045

Sex, Age; Pearson's Chi-squared test; MSI, TMB; Fisher's Exact test; PD-L1 (CPS), TMB; Wilcoxon-rank sum test. MSI, microsatellite instability; MSI-H, MSI-high; TMB, tumor mutation burden; PD-L1 CPS, programmed death-ligand 1 combined positive score.
